# An ultra-early biliary occlusion caused by a blood clot impaction inside a multi-hole covered self-expandable metal stent

**DOI:** 10.1055/a-2800-4701

**Published:** 2026-02-26

**Authors:** Tesshin Ban, Yoshimasa Kubota, Yota Hirayama, Kei Ando, Naoto Imura, Shun Sasoh, Takashi Joh

**Affiliations:** 1 36884 Department of Gastroenterology and Hepatology, Gamagori City Hospital, Gamagori, Japan

There is inconclusive evidence on whether to use an uncovered self-expandable metal stent (UC-SEMS) or a covered self-expandable metal stent (C-SEMS) for patients with unresectable or borderline distal malignant biliary obstruction (D-MBO 1 2 3 ). A new multi-hole C-SEMS (MH-C-SEMS), made of nitinol mesh covered by a silicone membrane with small 1.8-mm multiple holes, has been reported to have longer patency and prevents tumor ingrowth and stent migration compared to conventionally used UC-SEMS and C-SEMS 4 .

Herein, we reported a case of ultra-early stent occlusion caused by blood clot impaction on MH-C-SEM.

**Fig. 1:**
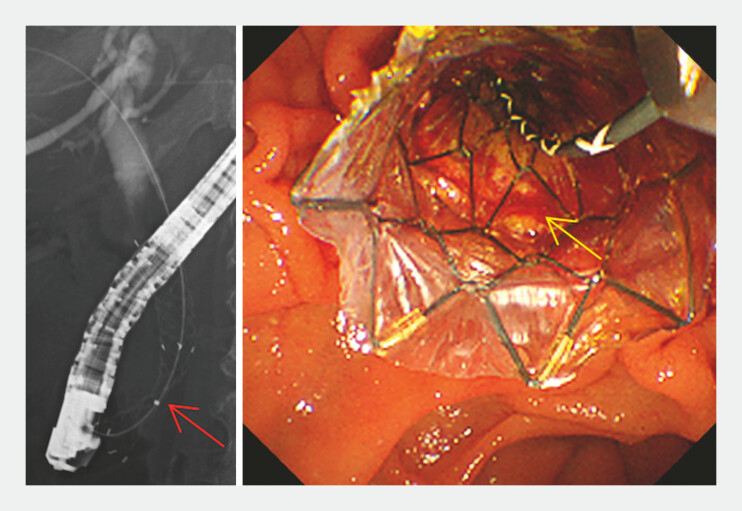
Fluoroscopic and endoscopic images of MH-C-SEMS in the distal malignant biliary obstruction. The red solid arrow indicates an incomplete expansion of MH-C-SEMS at the stricture, very close to the papilla. The yellow solid arrow indicates a slightly protruding tumor with blood staining through a hole in the stent membrane. MH-C-SEMS, multi-hole covered self-expandable metal stent.

**Fig. 2:**
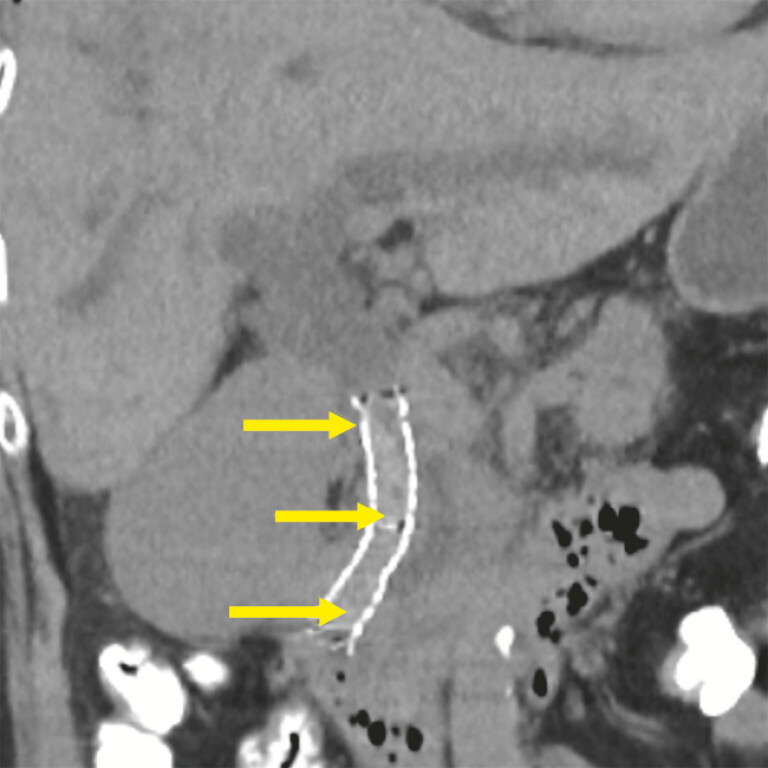
A computed tomography image taken 2 days after the index procedure. The yellow solid arrows indicate a high-density embolus suspected to be blood clot impaction along the entire length of the fully expanded MH-C-SEMS. MH-C-SEMS, multi-hole covered self-expandable metal stent.

**Fig. 3:**
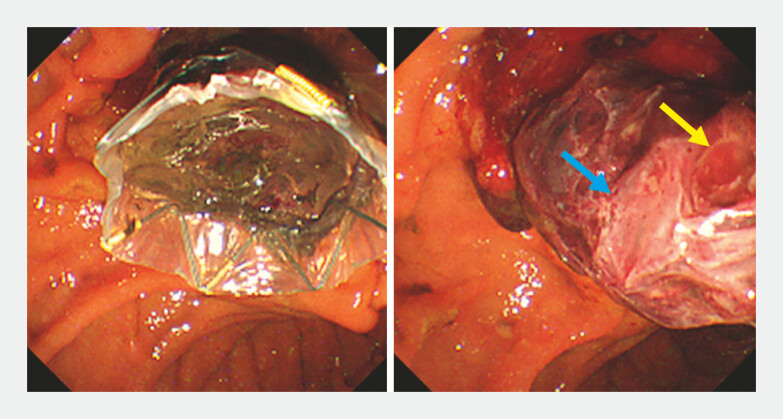
Endoscopic images of an impacted MH-C-SEMS and a withdrawn cast blood clot after stent removal. A round shape is carved into the cast blood clot ( yellow arrow) indicating that the tumor protrudes through a hole in the stent membrane. The stent wire stamped a rhombus shape (blue arrow) onto the blood clot. MH-C-SEMS, multi-hole covered self-expandable metal stent.

A 67-year-old woman with an unresectable, well-differentiated pancreatic adenocarcinoma in the groove region developed severe obstructive jaundice. She was scheduled to undergo chemotherapy; therefore, we performed transpapillary MH-C-SEMS placement across the papilla after sphincterotomy ( Fig. 1 , Video 1 ). However, she developed worsening jaundice and acute cholangitis. Two days after the index procedure, computed tomography imaging showed stent occlusion caused by blood clot impaction of MH-C-SEMS ( Fig. 2 ). When the initial MH-C-SEMS was removed with a snare, a cast blood clot shaped by the MH-C-SEMS appeared in the papilla ( Fig. 3 , Video 1 ). Therefore, we removed the blood clot using a balloon catheter, followed by the placement of double-pigtailed plastic stents ( Video 1 ). Her clinical course was uneventful, and she underwent chemotherapy.

Ultra-early biliary occlusion caused by blood clot impaction inside a multi-hole covered self-expandable metal stent.Video 1

In patients with D-MBO, the MH-C-SEMS has been shown to allow significantly longer stent patency (mean: 479 d; 95% confidence interval: 372–586 d) than the C-SEMS and UC-SEMS 4 . Stent dysfunction caused by blood clots occur in 1.9% of cases 5 . Additionally, hemorrhagic tumors or tumors involved in sphincterotomy could lead to blood oozing from uncovered holes, which can deposit acute blood clots on the stent membrane, potentially causing ultra-early stent occlusion. Physicians should consider this point if jaundice or cholangitis persists.

Endoscopy_UCTN_Code_CPL_1AK_2AD
